# Laser spectroscopy of indium Rydberg atom bunches by electric field ionization

**DOI:** 10.1038/s41598-020-68218-5

**Published:** 2020-07-23

**Authors:** A. R. Vernon, C. M. Ricketts, J. Billowes, T. E. Cocolios, B. S. Cooper, K. T. Flanagan, R. F. Garcia Ruiz, F. P. Gustafsson, G. Neyens, H. A. Perrett, B. K. Sahoo, Q. Wang, F. J. Waso, X. F. Yang

**Affiliations:** 1 Instituut voor Kern- en Stralingsfysica, KU Leuven, 3001 Leuven, Belgium; 2 School of Physics and Astronomy, The University of Manchester, Manchester, M13 9PL UK; 3 EP Department, CERN, 1211 Geneva 23, Switzerland; 4 Massachusetts Institute of Technology, Cambridge, MA 02139 USA; 5 Atomic, Molecular and Optical Physics Division, Physical Research Laboratory, Navrangpura, Ahmedabad, 380009 India; 6 School of Nuclear Science and Technology, Lanzhou University, Lanzhou, 730000 China; 7 Stellenbosch University, Merensky Building, Merriman Street, Stellenbosch, South Africa; 8 Photon Science Institute, Alan Turing Building, University of Manchester, Manchester, M13 9PY UK; 9 School of Physics and State Key Laboratory of Nuclear Physics and Technology, Peking University, Beijing, 100871 China

**Keywords:** Experimental nuclear physics, Electronic structure of atoms and molecules, Optical spectroscopy

## Abstract

This work reports on the application of a novel electric field-ionization setup for high-resolution laser spectroscopy measurements on bunched fast atomic beams in a collinear geometry. In combination with multi-step resonant excitation to Rydberg states using pulsed lasers, the field ionization technique demonstrates increased sensitivity for isotope separation and measurement of atomic parameters over previous non-resonant laser ionization methods. The setup was tested at the Collinear Resonance Ionization Spectroscopy experiment at ISOLDE-CERN to perform high-resolution measurements of transitions in the indium atom from the $\text {5s}^2\text {5d}\,^2\text {D}_{5/2}$ and $\text {5s}^2\text {5d}\,^2\text {D}_{3/2}$ states to $\text {5s}^2n$p $^2$P and $\text {5s}^2n\text {f}\,^2$F Rydberg states, up to a principal quantum number of $n=72$. The extracted Rydberg level energies were used to re-evaluate the ionization potential of the indium atom to be $46,670.107(4)\,\hbox {cm}^{-1}$. The nuclear magnetic dipole and nuclear electric quadrupole hyperfine structure constants and level isotope shifts of the $\text {5s}^2\text {5d}\,^2\text {D}_{5/2}$ and $\text {5s}^2\text {5d}\,^2\text {D}_{3/2}$ states were determined for $^{113,115}$In. The results are compared to calculations using relativistic coupled-cluster theory. A good agreement is found with the ionization potential and isotope shifts, while disagreement of hyperfine structure constants indicates an increased importance of electron correlations in these excited atomic states. With the aim of further increasing the detection sensitivity for measurements on exotic isotopes, a systematic study of the field-ionization arrangement implemented in the work was performed at the same time and an improved design was simulated and is presented. The improved design offers increased background suppression independent of the distance from field ionization to ion detection.

## Introduction

The ability to separate and study small quantities of isotopes from a large ensemble without losses is the limiting factor of many experimental studies in modern nuclear physics^1–4^, as exotic isotopes of interest can often only be produced at low rates (fewer than 100s of ions per second) and their accumulation into substantial quantities is prevented by their short half-lives. Furthermore, sensitive detection or separation of small quantities of isotopes also has numerous technological applications^5–11^. Fast beam collinear laser spectroscopy techniques have allowed high-precision measurements on short-lived isotopes, down to rates of fewer than 100 ions per second^3,12,13^. These approaches use the Doppler compression of an accelerated atomic beam to enable high-precision laser spectroscopy measurements to be performed in a collinear geometry^14,15^. This technique is now being implemented at radioactive ion beam facilities worldwide, giving a resolution of a few 10s of MHz, which is sufficient to resolve the hyperfine structure for nuclear physics studies^16–19^.

Motivated by a need for higher sensitivity to access exotic isotopes produced at rates lower than a few ions per second, a variation of the technique, the Collinear Resonance Ionization Spectroscopy (CRIS)^20,21^ experiment at CERN-ISOLDE^22^ has been developed. The technique is based on resonant laser excitation of atom bunches^23,24^ using a high-resolution pulsed laser, followed by resonant or non-resonant ionization of the excited atoms. The ions are then deflected away from the atoms which were not resonantly excited, allowing ion detection measurements with significantly reduced background. The experiment has so far reached a background suppression factor of $2.5\times 10^{8}$ providing a detection sensitivity down to yields of around 20 atoms per second^25^. The main source of ion background for the technique is due to collisional re-ionization of the atom beam (often also containing a substantial amount of isobaric contamination) with residual gas atoms along the collinear laser overlap volume. For this reason, considerable effort is given to reach ultra-high vacuum pressures ($<1\times 10^{-9}$mbar) in this laser-atom interaction region. The work reported here demonstrates that the incorporation of field ionization, previously tested on continuous atom beams^26–28^, can further increase the sensitivity of measurements on bunched atomic beams by also compressing the measurements into a narrow ionization volume. In addition, we show the approach has advantages for measurements of atomic parameters when combined with the multi-step pulsed narrow-band laser excitation. The sensitivity of the approach is further improved by removing the need for a powerful non-resonant laser ionization step, which often contributes substantially to the re-ionization background. This is due to non-resonant ionization of contaminant atomic species, which are often neutralised into excited atomic levels^29^, or can be excited by collisions into higher atomic levels^30^, that are easily ionized with powerful laser light. Using the field-ionization technique, high-resolution measurements were performed for the energies of the $\text {5s}^2n$p $^2$P and $\text {5s}^2n$f $^2$F Rydberg series (intermittently over principal quantum numbers $n=12$ to *n* = 72), which additionally enabled the re-evaluation of the ionization potential of the indium atom. The production of atom bunches was implemented using an ablation ion source^31^ with naturally abundant $^{113}$In and $^{115}$In isotopes.

In typical measurements using atomic transitions to extract nuclear structure parameters, both the upper and lower atomic states of the transition have a nuclear-structure dependence. However, due to the vanishing nuclear structure dependence of the Rydberg states^32,33^, the transition measurements made here allowed extraction of the nuclear structure dependent parameters of the lower atomic states alone. These measurements therefore allowed extraction of the hyperfine structure constants of the $\text {5s}^2$5d $^2\text {D}_{5/2}$ and $\text {5s}^2$5d $^2\text {D}_{3/2}$ states and their level isotope shifts (LISs), the shift in the level energies between the $^{113,115}$In isotopes. This allows a direct comparison to atomic calculations of the isotope shift contribution from a single atomic state, in contrast to typical atomic transitions measurements where upper and lower state contributions are combined. The experimentally determined LISs, hyperfine structure constants and ionization potential of the indium atom are compared to calculations from relativistic coupled-cluster theory^34^. Comparisons with direct measurements of atomic states provide a valuable benchmark to assist the extraction of nuclear structure observables with high accuracy^31,35^. In addition, the development of high-accuracy atomic calculations has the potential to probe new aspects of the atomic nucleus and its interactions^36–41^.

## Methods

### Experimental setup

**Figure 1:**
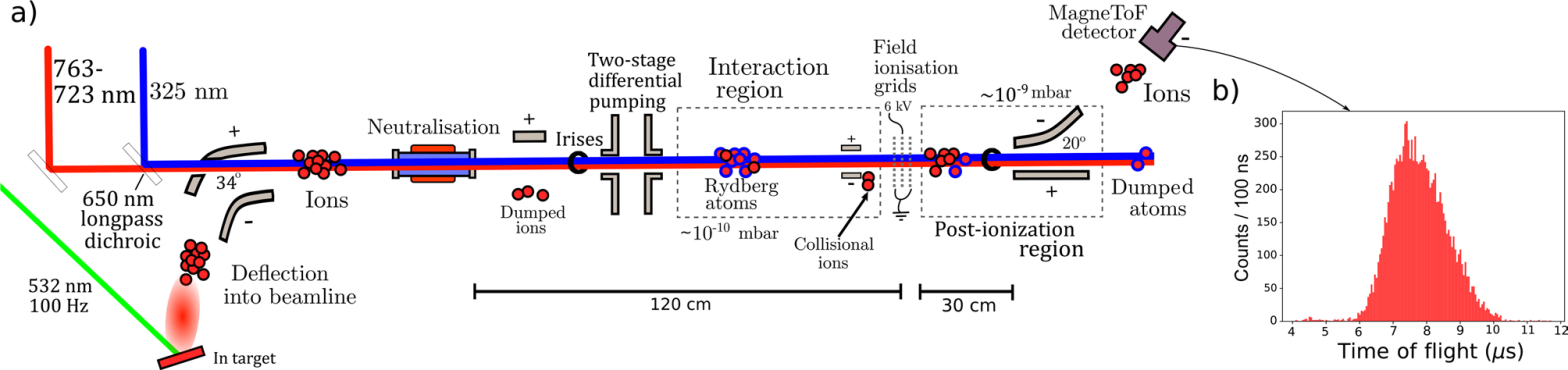
(**a**) A schematic diagram of the layout of the modified CRIS experiment, to use field ionization. Electrostatic deflectors guide the ions created by ablation to be neutralised. Atoms are excited to Rydberg states by amulti-step resonant laser scheme, and are subsequently field ionized and counted by an ion detector. (**b**) The time-of-flight distribution of the bunch created by the ablation ion source. Figure created using Refs.^44,45^.

The measurements reported here were performed following a modification of the CRIS experimental setup^42,43^. A schematic layout of the modified setup is shown in Fig. 1a. In$^+$ ions were produced using an ablation ion source (detailed in Refs.^31,42^), with a pulsed 532-nm Litron LPY 601 50-100 PIV Nd:YAG laser focused to produce a fluence of >0.5 J/cm$^2$ on a solid indium target (99% purity).

This produced high-intensity bunches of indium ions at the 100 Hz repetition rate of the laser with a typical bunch width of $2.0(3)\,\upmu \hbox {s}$ (see Fig. 1b). This ablation laser was used as the start trigger to synchronise the atomic bunches in the interaction region with the pulsed lasers subsequently used for spectroscopy. A set of ion optics were used to focus and accelerate the In$^+$ ions to 25 keV and deflect them by 34$^\circ$ to overlap with two laser beams in a collinear geometry. The acceleration creates a kinematic separation in the transition frequency of the naturally abundant isotopes $^{115}$In (95.72%) and $^{113}$In (4.28%), which greatly enhances the isotope selectivity compared to in-source laser spectroscopy separation or measurement techniques^5,46^, laser induced breakdown spectroscopy (LIBS)^47^ or in-gas cell laser ionization spectroscopy (IGLIS)^48^. Collinear ionization techniques can therefore also be used to supplement^49^ the isotope selectivity of conventional mass spectrometry techniques^50,51^.

The ions were subsequently neutralised, using a sodium-filled charge-exchange cell heated to 300(10)$^\circ$C, with an efficiency of 60(10)%, where 64% of the atomic population is simulated to be in the $\text {5s}^2$5p $^2\text {P}_{3/2}$ metastable state^29^. The ions which were not neutralised were deflected electrostatically following the cell. After approximately 80 cm of flight (the maximum 4 $\upmu \mathrm{s}$ bunch width corresponds to a spatial spread of 81 cm at 25 keV), the indium atoms were then excited using either the $\text {5s}^2$5p $^2\text {P}_{3/2}$ $\rightarrow$ $\text {5s}^2\text {5d}\,^2\text {D}_{5/2}$ (325.6 nm) or $\text {5s}^2$5p $^2\text {P}_{3/2}$ $\rightarrow$ $\text {5s}^2\text {5d}\,^2\text {D}_{3/2}$ (325.9 nm) transition, depending on the Rydberg series to be studied.

**Figure 2:**
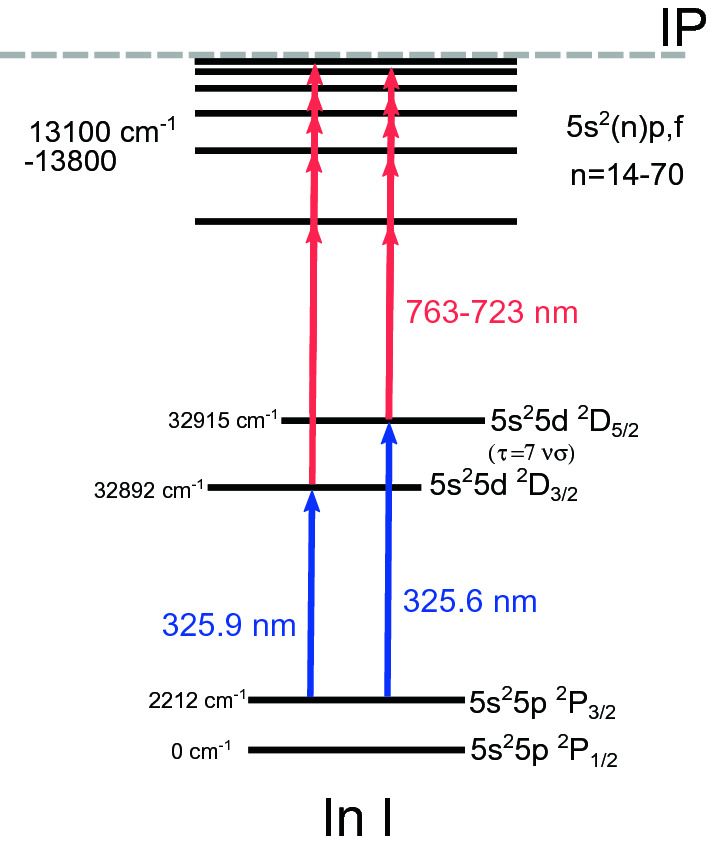
The multi-step laser excitation schemes used in this work on the indium atom. Field ionization was used following the two laser steps to a Rydberg state. The lifetime of the intermediate $\text {5s}^2$5d $^2\text {D}_{5/2}$, $\text {5s}^2$5d $^2\text {D}_{3/2}$ states is 7.0(4) ns^52^. Figure created using Ref.^44^.

The multi-step ionization schemes used in this work are shown in Fig. 2. The first step light was produced using a Spectron Spectrolase 4000 pulsed dye laser with DCM dye dissolved in ethanol. This produced fundamental light at 650 nm which was frequency doubled using a BaB$_2$O$_4$ crystal to 325 nm. The linewidth of the laser was $\sim$14 GHz, allowing excitation of all of the hyperfine structure components of the $\text {5s}^2$5p $^2\text {P}_{3/2}^o$ state, while the 699 GHz separation between the $\text {5s}^2$5d $^2\text {D}_{5/2}$ and $\text {5s}^2$5d $^2\text {D}_{3/2}$ states required tuning the laser to each fine structure transition. The dye laser was pumped with 532-nm light from the second output head of the Litron LPY 601 50-100 PIV Nd:YAG laser used for ablation. The second excitation step was scanned in laser frequency to perform high-resolution laser spectroscopy, from either the $\text {5s}^2$5d $^2\text {D}_{5/2}$ or $\text {5s}^2$5d $^2\text {D}_{3/2}$ state to a Rydberg state of the $\text {5s}^2n$f or $\text {5s}^2n$p series, ranging over *n* = 14–72 (763–723 nm). The high-resolution infrared laser light was produced using an injection-locked Ti:Sapphire laser^53,54^ pumped by a LEE LDP-100MQ Nd:YAG laser and seeded using a narrowband continuous-wave Sirah Matisse Ti:Sapphire laser. This provided the pulsed narrowband (20(5) MHz^54^) laser light to be used for spectroscopy. The resonantly excited indium Rydberg states were then field ionized in a longitudinal geometry by thin wire grids with a field gradient of $7.5\,\mathrm{kV\,cm}^{-1}$, detailed in the following section. Spatial alignment of the atom and ion paths was performed using irises and Faraday cups. The ion beam waist was measured to be around 3(1) mm using an iris^42^, below this a reduction in beam current begin to be observed. This was measured $\sim$30 cm from the neutralisation cell. Following ionization the ions were deflected by 20$^\circ$ onto a ETP DM291 MagneTOF^TM^ detector and the recorded count rate was used to produce the hyperfine spectra as a function of the infared laser frequency. The wavelengths were measured using a HighFinesse WSU2 wavemeter. This was calibrated and drift stabilized by simultaneous measurement of a Toptica DLC DL PRO 780 diode laser locked to the $\text {5s}^2$S$_{1/2}$ $\rightarrow$ 5p $^2\text {P}_{3/2}$ F = 2 - 3 transition of $^{87}$Rb using a saturated absorption spectroscopy unit^55^.

### Field ionization using wire grids

**Figure 3:**
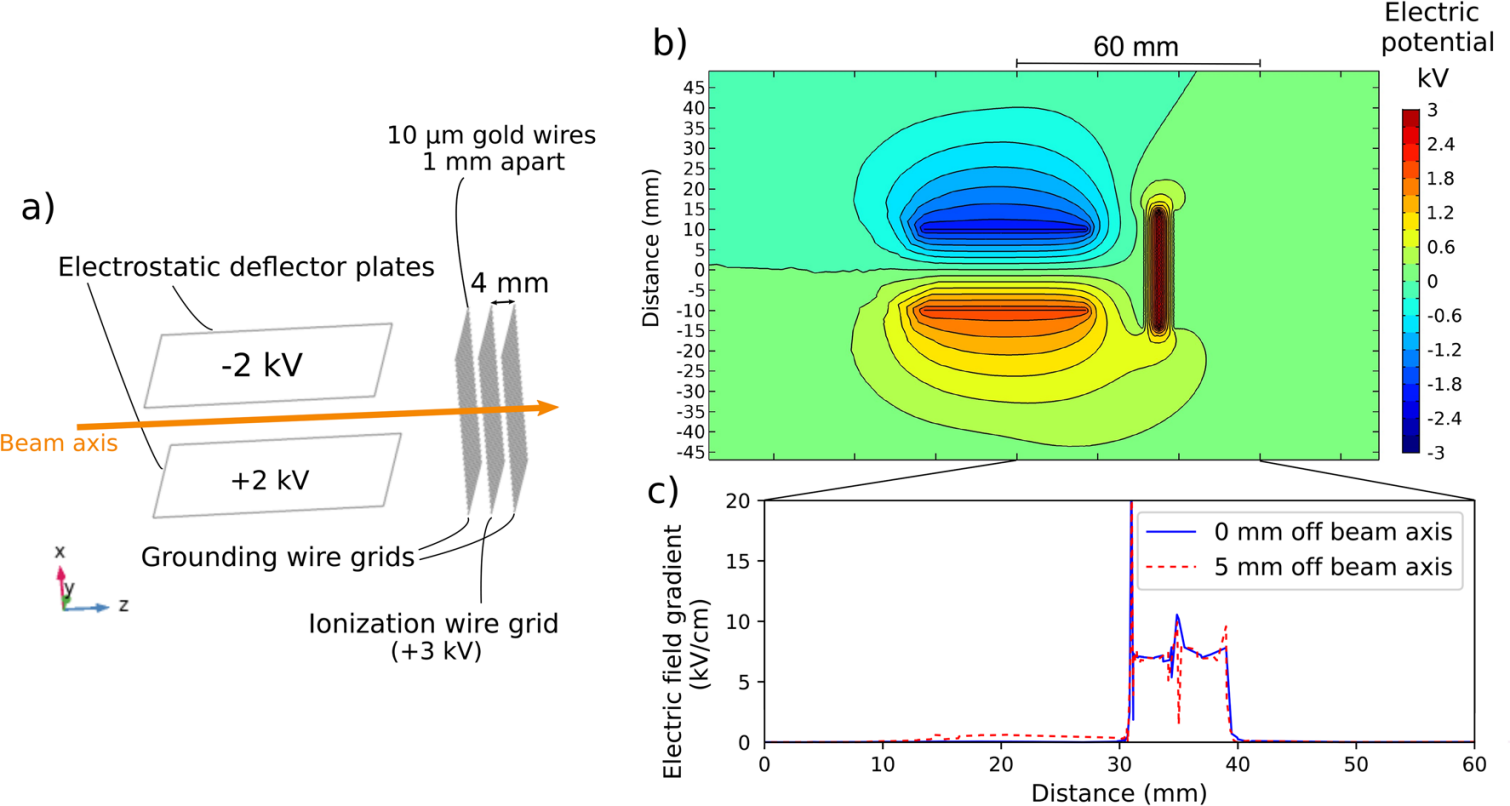
(**a**) A schematic of the electrode arrangement used in this work (**b**) The electric potential of the field-ionization arrangement used for the measurements of this work, and (**c**) the accompanying electric field gradient, simulated from measurements of voltages applied during this work. Figure created using Refs.^44,45,56^.

The field ionization of the Rydberg states in this work was performed using the electrode arrangement shown in Fig. 3, located in the position indicated in Fig. 1. Three consecutive grids of parallel gold wires, of $10\,\upmu \hbox {m}$ thickness and with 1 mm separation between wires were used as electrodes to create the field for ionization, as shown in Fig. 3a,b. The outmost grids were used to provide ground shielding. The wire grids were mounted on a printed circuit board and spaced 4 mm apart, resulting in an average electric field gradient of $7.5\,\mathrm{kV\,cm}^{-1}$ for the 3 kV potential applied to the innermost grids. The arrangement included two parallel electrostatic deflector plates with opposing polarity before the grids, in order to deflect background ions. These background ions originate from non-resonant processes in the preceding 120 cm flight path between the charge exchange cell and the field-ionization grids, which is referred to as the ‘laser-atom interaction region’. The region surrounding the grids is further called the ‘field-ionization region’, and the region between the last field-ionization grid and the ion detector will be referred to as the ‘post-ionization region’ (Fig. 1).

This longitudinal electric field-ionization arrangement was chosen so the closely spaced grids could provide a small ionization volume of $0.4\,\hbox {cm}^{3}$ with a very well defined electric field gradient. This small volume has to be compared to the $120\,\hbox {cm}^{3}$ volume where non-resonant laser ionization would normally take place. This is a reduction by a factor of 300 in volume in which collisional ionization of the atom beam can occur with residual gases. This is a substantial source of background which can be removed when field ionization is used. However, the background reduction using this setup is limited by the distance between field ionization and the ion detection, as this distance can also contribute to an indistinguishable collisional background. This is shown in a systematic study of the setup and an improved design discussed in the Section “An improved field-ionization setup”. This section follows the discussion of the spectroscopy results obtained using the setup described above. An additional advantage of the grid field ionization arrangement is that the grids approximate a plane geometry for the electric field, removing the dependence on transverse displacement of the atom beam on the field gradient experienced by the atoms (see Fig. 3c) compared to compared to traditional tube field-ionization geometries where large differences in electric field gradient can be found transverse to the beam axis^28,57^. The electrostatic simulations were performed using COMSOL Multiphysics.

### Coupled-cluster calculations

In order to compare to our experimental results, the indium atom ionization potential, hyperfine structure constants $\text {A}_{\text {hf}}$ and $\text {B}_{\text {hf}}$, and atomic isotope shift factors were calculated using relativistic coupled-cluster (RCC) theory as outlined below. The $\text {A}_{\text {hf}}$ and $\text {B}_{\text {hf}}$ constants were calculated using an expectation-value evaluation approach as described in Ref.^58^. While the atomic parameters for the isotope shift were calculated using an analytic response approach (AR-RCC), an approach developed for increased accuracy for the evaluation of isotope shift contributions, as described in Ref.^35^.

In RCC theory, the wave function ($\vert \Psi _v \rangle$) of an atomic state with a closed-core and a valence orbital *v* can be expressed as

1 $$\begin{aligned} \vert \Psi _v \rangle = e^T [ 1+ S_v ] \vert \Phi _v \rangle , \end{aligned}$$

where $\vert \Phi _v \rangle$ is the mean-field wave function, defined as $\vert \Phi _v \rangle =a_v^{\dagger } \vert \Phi _0 \rangle$, with the Dirac-Hartree-Fock (DHF) wave function of the closed-core, $\vert \Phi _0 \rangle$. Here, *T* and $S_v$ are the RCC excitation operators which incorporate electron correlation effects by exciting electrons in $\vert \Phi _0 \rangle$ and $\vert \Phi _v \rangle$, respectively, to the virtual space. The amplitudes of the RCC operators and energies were obtained by solving the following equations

2 $$\begin{aligned} \langle \Phi _K \vert \left( H e^T \right) _c \vert \Phi _0 \rangle= & {} \delta _{K,0} E_0 \; , \end{aligned}$$

and

3 $$\begin{aligned} \langle \Phi _L \vert \left( H e^T \right) _c S_v + \left( H e^T \right) _c \vert \Phi _v \rangle= & {} \left( \delta _{L,0} + \langle \Phi _L \vert S_v \vert \Phi _v \rangle \right) E_v \; , \end{aligned}$$

where *H* is the atomic Hamiltonian, and $\vert \Phi _K \rangle$ and $\vert \Phi _L \rangle$ denote the excited determinants with respect to $\vert \Phi _0 \rangle$ and $\vert \Phi _v \rangle$. Here, $E_0$ and $E_v$ correspond to the energies of the closed-core and the closed-core with the valence orbital respectively. Thus, the difference between $E_v$ and $E_0$ gives the binding energy or the negative of the ionization potential (IP) of the electron from the valence orbital, *v*. The hyperfine structure constants of the unperturbed state were evaluated by

4 $$\begin{aligned} \frac{\langle \Psi _v | O | \Psi _v \rangle }{\langle \Psi _v| \Psi _v \rangle }= & {} \frac{\langle \Phi _v | [1+ S_v^{\dagger }] e^{T^{\dagger }} O e^T [1+S_v] | \Phi _v \rangle }{\langle \Phi _v| [1+ S_v{\dagger }] e^{T^{\dagger }} e^T [1+S_v] | \Phi _v \rangle } , \end{aligned}$$

where *O* is the hyperfine interaction operator. In the above expression, the non-terminating series of $e^{T^{\dagger }} O e^T$ and $e^{T^{\dagger }} e^T$ in the numerator and denominator, respectively, were calculated by adopting a self-consistent iterative procedure as described in Ref.^58^. All-possible singles and doubles excitations were included in the RCC calculations (RCCSD) methods, by defining

5 $$\begin{aligned} T=T_1 + T_2 \ \ \ \ \text {and} \ \ \ \ S_v=S_{1v} + S_{2v} \end{aligned}$$

with subscripts 1 and 2 representing level of excitation, for determining the energies and hyperfine structure constants. The calculations were performed by first considering the Dirac-Coulomb (DC) Hamiltonian, then including the Breit and lower-order quantum electrodynamics (QED) interactions as described in Ref.^59^. Corrections due to the Bohr-Weisskopf (BW) effect to the hyperfine structure constants were estimated by considering a Fermi-charge distribution of the nucleus.

The AR-RCC approach adopted to determine the field shift (FS), normal mass shift (NMS) and the specific mass shift (SMS) constants was implemented by evaluating the first order perturbed energies due to the respective operators, as discussed in Ref.^35^. The AR-RCC theory calculations were also truncated using the singles and doubles excitation approximation (AR-RCCSD) when used to calculate the FS, NMS and SMS constants in this work. Contributions from the DC Hamiltonian and corrections from the Breit and QED interactions were evaluated explicitly and are shown in Table 2.

## Analysis and results

**Figure 4:**
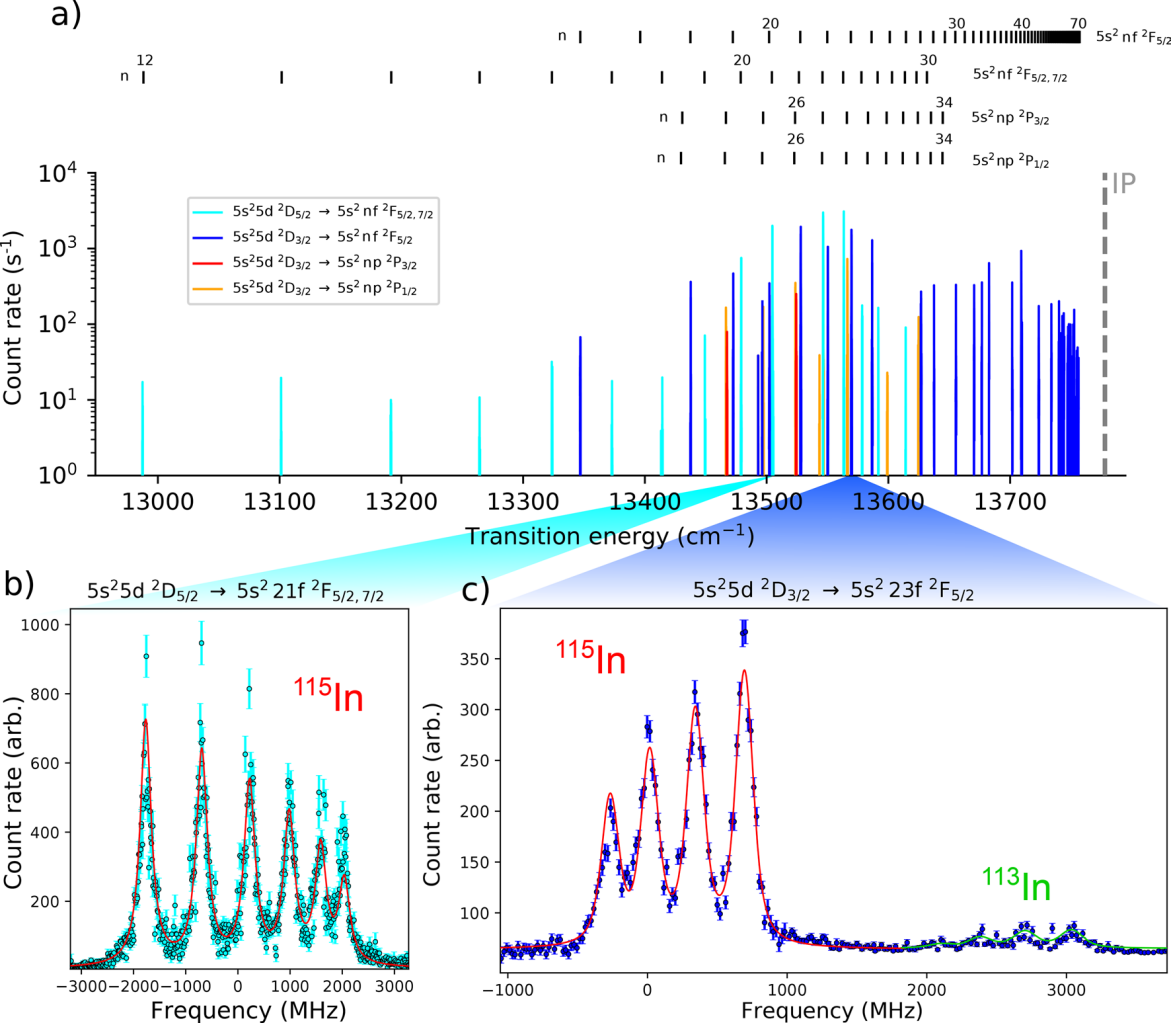
Summary of the high-resolution measurements of the transitions to Rydberg states from the $^2\text {P}_{1/2}$ $\text {5s}^2n$p, $^2\text {P}_{3/2}$ $\text {5s}^2n$p and $^2\text {F}_{5/2}$ $\text {5s}^2n$f series, showing (**a**) the spectrum of resonances measured in the 770-720 nm range, with an inset in black indicating energies for the members of the Rydberg series using Expression 6. Example hyperfine spectra of the (**b**) $\text {5s}^2$5d $^2\text {D}_{5/2}$ and c) $\text {5s}^2$5d $^2\text {D}_{3/2}$ lower states. Fits to the spectra of $^{115}$In are indicated in red. Figure created using Refs.^44,45^. A fit to $^{113}$In is indicated in green.

A summary of the high-resolution Rydberg series measurements is presented in Fig. 4. A range of wavelengths from 720 to 770 nm (14000–12900 cm$^{-1}$) were used to cover the transitions to Rydberg states in this work (*n* = 12-72), as shown in Fig. 4a. The energies of the states in the Rydberg series (vertical black dashes in Fig. 4a) were estimated using the Rydberg formula^60^ extrapolating from the energies of the five lowest principal quantum number atomic states (*n* = 4-9 for *n*f, *n* = 5–10 for *n*p), taken from literature^61^. See the Section “Evaluation of the ionization potential of the indium atom” for details. Figure 4b,c show hyperfine spectra obtained for the $\text {5s}^2$5d $^2\text {D}_{5/2}$ $\rightarrow$ $\text {5s}^2n$f $^2\text {F}_{5/2, 7/2}$ and $\text {5s}^2$5d $^2\text {D}_{3/2}$ $\rightarrow$ $\text {5s}^2n$f $^2\text {F}_{5/2}$ transitions respectively. The hyperfine structure resulting from the $\text {5s}^2$5d $^2\text {D}_{5/2}$ and $\text {5s}^2$5d $^2\text {D}_{3/2}$ states is visible in these spectra, while the contribution from the Rydberg state in both cases is vanishingly small due to the reduced overlap of the electronic wavefunctions at the nucleus^32,33^. The fine structure splitting between $\text {5s}^2n$f $^2\text {F}_{5/2}$ and $\text {5s}^2n$f $^2\text {F}_{7/2}$ Rydberg states has been measured to be <1 MHz^62^ and smaller than the linewidth of the laser used in this work. The upper states of transitions from $\text {5s}^2$5d $^2\text {D}_{5/2}$ are therefore denoted as $\text {5s}^2n$f $^2\text {F}_{5/2,7/2}$ to indicate that excitation to both the $\text {5s}^2n$f $^2\text {F}_{5/2}$ and $\text {5s}^2n$f $^2\text {F}_{7/2}$ states are included.

### Hyperfine structure constants and isotope shifts

The extracted magnetic dipole and electric quadrupole hyperfine constants, $\text {A}_{\text {hf}}$ and $\text {B}_{\text {hf}}$, of the $\text {5s}^2$5d $^2\text {D}_{5/2}$ and $\text {5s}^2$5d $^2\text {D}_{3/2}$ states for $^{113,115}$In are displayed in Table 1. The constants were determined by least-square minimisation fitting^63^ of the obtained hyperfine spectra to the well known hyperfine structure relations^64^ with $\text {A}_{\text {hf}}$ and $\text {B}_{\text {hf}}$ as free parameters. A Voigt line profile^65^ was used in the fitting with the Gaussian and Lorentzian components and transition intensities as free parameters. The ablation ion source was measured^31^ to produce ions with energy spreads of <10 eV (<100 MHz at 25 keV), however the energy distribution is not necessarily fully Gaussian. This likely accounts for the slight ‘under-fitting’ of the amplitude of the peaks of the hyperfine spectra, visible in Fig. 4b,c. This is evident in the Lorentzian component of 200(100) MHz (compared to the laser linewidth of 20(5) MHz), as the Lorentzian profiles have a lower peak amplitude for the same FWHM compared the Gaussian profiles, the increased Lorentzian component may compensate for the non-Gaussian part of the ablation ion energy spread. The <0.5 mJ/cm$^2$ fluences of the 760-720 nm transitions to the Rydberg states are expected to contribute negligibly to changes of the resonance centroids compared to the AC Stark shift introduced from temporally overlapping high-power lasers for non-resonant ionization^55^.

The presented $\text {A}_{\text {hf}}$ and $\text {B}_{\text {hf}}$ values are an average of the results from well-resolved hyperfine spectra obtained in this work, over principal quantum numbers *n* = 15-34. For Rydberg states below $n\approx$20, the applied laser power saturated the transition and this led to power broadening which reduced the resolution of those hyperfine spectra. The transitions above $n\approx$30 were not saturated (see Section “Systematics of the field-ionization setup”), because the transition probability scales approximately^66^ as $n^{-3}$. For these transitions a greater ablation laser fluence and ion source extraction potential were required to obtain a similar resonant signal level. This resulted in an increased energy spread of the ion bunch, increasing the Gaussian contribution to the linewidth to greater than 100 MHz and obscuring the hyperfine spectra in those cases. No statistically significant deviation was seen for contributions from the Rydberg states to the hyperfine structure constants down to *n* = 12. The larger uncertainty of the extracted $\text {B}_{\text {hf}}$ values was due to their small magnitudes compared to the laser linewidth of 20(5) MHz. The larger uncertainty on the $^{113}$In values was due to the lower statistics, related to its lower natural abundance of 4.28%^67^, in combination with a reduction in excitation efficiency due to the 14-GHz linewidth of the dye laser used for the first step transition which was centered at a frequency for $^{115}$In. An example of the spectra of $^{113}$In relative to $^{115}$In is shown in Fig. 4c. The isotope shifts for the levels (LIS) were also extracted and are displayed in Table 1. The LIS in this work is defined as the difference in the transition energy, $\Delta E$, of isotopes $^{115}$In and $^{113}$In, LIS = $\Delta E_{113}-\Delta E_{115}$, for the transitions from the $\text {5s}^2$5d $^2\text {D}_{5/2}$ or $\text {5s}^2$5d $^2\text {D}_{3/2}$ state to Rydberg states, $\Delta E = E_{\mathrm {RB}}-E_{\mathrm {D_{5/2,3/2}}}$. The $E_{\mathrm {RB}}$ terms cancel due to their negligible change between the isotopes^32,33^. The result therefore being the isotope shift of the $\text {5s}^2$5d $^2\text {D}_{5/2}$ or $\text {5s}^2$5d $^2\text {D}_{3/2}$ energy levels.

**Table 1:** Hyperfine structure constants and LISs of the $\text {5s}^2\text {5d}\,^2\text {D}_{5/2}$ and $\text {5s}^2\text {5d}\,^2\text {D}_{3/2}$ states measured for $^{113,115}$In.

State		$^{115}$In		$^{113}$In		
		$\text {A}_{\text {hf}}$ (MHz)	$\text {B}_{\text {hf}}$ (MHz)	$\text {A}_{\text {hf}}$ (MHz)	B $_{\text {hf}}$ (MHz)	LIS $^\ddagger$ (MHz)
$5 {\text{s}}^2$5d $^2\text {D}_{5/2}$	**Exp. **	**151.2(9)**	**33(20)**	**151(6)**	**28(200)**	−**103(50)**
	**Theor.**					
	DHF	1.87	2.53	1.86	2.50	
	RCCSD	39.66	24.84	39.57	24.53	
	$+$Breit	39.80	24.84	39.71	24.53	
	$+$QED	40.00	24.99	39.91	24.68	
	$+$BW	**39.87**	**25.01**	**39.78**	**24.70**	$-{\mathbf 67.3(3.2)} ^\dagger$
$5 {\text {s}}^2$5d $^2\text {D}_{3/2}$	**Exp. **	−**64(1)**	**41(20)**	−**68(5)**	**20(60)**	−**98(30)**
	**Theor.**					
	DHF	4.37	1.83	4.36	1.81	
	RCCSD	− 9.89	17.82	− 9.87	17.60	
	$+$Breit	− 9.89	17.81	− 9.87	17.59	
	$+$QED	− 9.87	17.92	− 9.85	17.70	
	$+$BW	−**9.74**	**17.98**	−**9.72**	**17.72**	−**67.7(3.2)**$^\dagger$

$^\dagger$Theoretical LISs were calculated using the F, $\text {K}_{\text {SMS}}$ and $\text {K}_{\text {NMS}}$ constants from Table 2 obtained from the AR-RCCSD approach, combined with the experimentally measured change in root-mean-square charge radius, $\delta \left\langle r^2 \right\rangle _{\mu }^{113, 115}=-0.157(11)$ fm$^2$, taken from Ref.^68^, which gave the LIS uncertainty for the ‘calculated’ values in the table shown in brackets. Bold text indicate the final reported experimental and theoretical values.

The calculated $\text {A}_\text {hf}$ and $\text {B}_\text {hf}$ values of the $\text {5s}^2\text {5d}\,^2\text {D}_{3/2}$ and $\text {5s}^2\text {5d}\,^2\text {D}_{5/2}$ states from the RCCSD method are presented in Table 1.

Literature nuclear magnetic dipole moment values of $\mu$ = 5.5289(2) $\mu _N$ (Refs.^69,70^) and nuclear electric quadrupole moment values of *Q* = 0.767(11) b (Ref.^31^) for $^{113}$In, and of $\mu$ = 5.5408(2) $\mu _N$ (Refs.^70,71^) and *Q* = 0.781(7) b (Ref.^31^) for $^{115}$In were used to evaluate the $\text {A}_\text {hf}$ and $\text {B}_\text {hf}$ values in Table 1, from the calculated quantities of $\text {A}_\text {hf}I$/$\mu$ and $\text {B}_\text {hf}$/*Q*.

The DHF values of $\text {A}_{\text {hf}}$ were calculated to be 4.37 MHz and 1.87 MHz, whereas the RCCSD calculations gave − 9.74 MHz and 39.87 MHz compared to the experimental values − 64(2) MHz and 151.2(9) MHz for the $\text {5s}^2$5d $^2\text {D}_{3/2}$ and $\text {5s}^2$5d $^2\text {D}_{5/2}$ states of $^{115}$In, respectively. The $\text {B}_\text {hf}$ values are within the 1$\sigma$ uncertainty of the experimental results, although the experimental uncertainty was large. The difference in the BW correction to the $\text {A}_\text {hf}$ values between $^{113}$In and $^{115}$In were found to be negligibly small (<0.01 MHz). Contributions from Breit and QED interactions were also found to be small. This indicates that electron correlations due to core-polarization effects play the principal role in bringing the results close to the experimental values. Thus, the experimental $\text {A}_{\text {hf}}$ values deviate in contrast to the LIS calculations using the AR-RCCSD calculations at the same level of truncation to singles and doubles excitation. An explanation demands including triples excitations or employing a more rigorous theoretical approach for the evaluation of $\text {A}_{\text {hf}}$ factors. This indicates that the behaviour of electron correlations can be very different in the determination of $\text {A}_{\text {hf}}$ and $\text {B}_{\text {hf}}$ values, which has also been identified previously in e.g. Ref.^72^. It is known that electron correlations, mainly the all-order core-polarization effects arising through $OS_{2v}$ and its complex conjugate terms, can contribute more than 100% in high-precision calculations of $\text {A}_{\text {hf}}$ values in the $D_{5/2}$ states of alkali-like atomic systems^73,74^. We find that the higher-order core-polarization effects, embedded in the $S_{2v}^{\dagger } O S_{2v}$ RCC term, are the greatest contribution in the evaluation of $\text {A}_{\text {hf}}$ values of both the $\text {5s}^2\text {5d}\,^2\text {D}_{3/2}$ and $\text {5s}^2\text {5d}\,^2\text {D}_{5/2}$ states in the indium isotopes. These effects can be enhanced further with the inclusion of triple excitation configurations in the RCC method. We, therefore, anticipate that much better agreement between the experimental and theoretical results for the $\text {A}_{\text {hf}}$ values can be obtained by incorporating these higher-level electronic configurations in the RCC calculations, which we defer to the future work. In Table 1, a comparison is also made between calculated LIS values with the measurements for the $\text {5s}^2$5d $^2\text {D}_{3/2}$ and $\text {5s}^2$5d $^2\text {D}_{5/2}$ states. The calculated FS (F), NMS ($\text {K}_\text {NMS}$) and SMS ($\text {K}_\text {SMS}$) constants, used to determine the calculated LIS values, are reported in Table 2 along with the included corrections. For comparison to our relativistic *ab-initio* calculations of $\text {K}_\text {NMS}$, the $\text {K}_\text {NMS}$ constant values from the non-relativistic approximation are also shown, estimated by the relation $\text {K}_\text {NMS}=E_i m_e$ and experimental energies^61^, $E_i$. Unlike the $\text {A}_\text {hf}$ hyperfine structure constants, we find a good agreement between the measured and theoretical values for the LISs of the $\text {5s}^2$5d $^2\text {D}_{3/2}$ and $\text {5s}^2$5d $^2\text {D}_{5/2}$ states by substituting the calculated IS constants.

**Table 2:** Calculated F, $\text {K}_{\text {SMS}}$ and $\text {K}_{\text {NMS}}$ constants of the $\text {5s}^2$5d $^2\text {D}_{5/2}$ and $\text {5s}^2$5d $^2\text {D}_{3/2}$ states for the In atom using DHF and AR-RCCSD approaches. Experimental level energies for the non-relativistic $\text {K}_\text {NMS}=E_i m_e$ approximation were taken from Ref.^61^. The factors were used to calculate the LIS values given in Table 1, using the expression LIS = F$\delta \left\langle r^2 \right\rangle _{\mu }^{113, 115}$ + $\mu$($\text {K}_{\text {NMS}}$ + $\text {K}_{\text {SMS}}$), where $\mu$ = (m$_{113}$-m$_{115}$)/(m$_{113}$m$_{115}$) is the mass modification factor using atomic masses from Ref.^75^

State	Method	F (MHz/fm$^2$)	$\text {K}_{\text {NMS}}$ (GHz amu)	$\text {K}_{\text {SMS}}$ (GHz amu)
$\text {5s}^2$5d $^2\text {D}_{5/2}$	DHF	$\sim 0.0$	277.95	− 38.48
	**AR-RCCSD**	293.33	198.76	− 55.92
	$+$Breit	293.56	198.72	− 55.41
	$+$QED	288.39	**198.97**	− 56.41
	**Exp.**		**226.20777(13)**	
$\text {5s}^2$5d $^2\text {D}_{3/2}$	DHF	$\sim 0.0$	277.78	− 37.39
	**AR-RCCSD**	295.99	198.59	− 55.69
	$+$Breit	296.19	198.54	− 55.22
	$+$QED	291.00	**198.88**	− 56.28
	**Exp.**		**226.59111(13)**	

Bold text indicate the final reported experimental and theoretical values.

### Rydberg state energies

In order to reduce the systematic error of the measured transition frequencies, reference scans were performed every few hours using transitions to the $\text {5s}^2$21f $^2\text {F}_{5/2}$ state. When the $\text {5s}^2$5p $^2\text {P}_{3/2}$ $\rightarrow$ $\text {5s}^2\text {5d}\,^2\text {D}_{3/2}$ first step transition was used, this was performed using the $\text {5s}^2$5d $^2\text {D}_{3/2}$ $\rightarrow$ $\text {5s}^2$21f $^2\text {F}_{5/2}$ transition. While for the $\text {5s}^2$5p $^2\text {P}_{3/2}$ $\rightarrow$ $\text {5s}^2\text {5d}\,^2\text {D}_{5/2}$ first step transition, the $\text {5s}^2$5d $^2\text {D}_{5/2}$ $\rightarrow$ $\text {5s}^2$21f $^2\text {F}_{5/2,7/2}$ transition was used. This allowed measurements of the Rydberg series to be referenced to the same $\text {5s}^2$21f $^2\text {F}_{5/2}$ state. The absolute energy of the $\text {5s}^2$21f $^2\text {F}_{5/2}$ state was determined for the first time in this work, using an average of measurements of the $\text {5s}^2$5d $^2\text {D}_{5/2}$ $\rightarrow$ $\text {5s}^2$21f $^2\text {F}_{5/2, 7/2}$ and $\text {5s}^2$5d $^2\text {D}_{3/2}$ $\rightarrow$ $\text {5s}^2$21f $^2\text {F}_{5/2}$ transition energies, combined with literature values for the $\text {5s}^2$5d $^2\text {D}_{5/2}$ and $\text {5s}^2$5d $^2\text {D}_{3/2}$ states, taken from Ref.^76^. This gave an averaged value of 46420.309(6) cm$^{-1}$ (1,391,645,846(175) MHz) for the energy of the $\text {5s}^2$21f $^2\text {F}_{5/2}$ state, as presented in Table 3. The 150 MHz uncertainty from the literature values was the largest contribution to the final uncertainty. Other sources of systematic uncertainty to the absolute energy measured for the $\text {5s}^2$21f $^2\text {F}_{5/2}$ state are also presented in Table 3. $\sigma (\nu )$ is the uncertainty arising from measurement of the $\text {5s}^2$5d $^2\text {D}_{3/2}$ $\rightarrow$ $\text {5s}^2$21f $^2\text {F}_{5/2}$ and $\text {5s}^2$5d $^2\text {D}_{3/2}$ $\rightarrow$ $\text {5s}^2$21f $^2\text {F}_{5/2}$ transition frequencies, which includes the statistical uncertainty, uncertainty in the fitting and variations in beam energy. $\sigma (\text {T}_\text {B})$ is the systematic uncertainty in the beam energy, provided by a Heinzinger PNChp 30000-5 power supply, which has an accuracy of <0.02$\%$ (5 V at 25 keV) and a quoted stability of <0.001$\%$ (250 mv) over 8 hours or per $1^{\circ }$C. The extraction electric potential at the point of creation of the ablation ions inside the source also contributes to an uncertainty in the beam energy, $\sigma$ Ext. The largest electric potential was found to be 10 v in simulations of the ion source^31^. $\sigma (\lambda )$ is the manufacture quoted absolute accuracy of the HighFinesse WSU2 wavemeter used. The $\sigma (\text {T}_\text {B})$, $\sigma$ Ext. and $\sigma (\lambda )$ systematic uncertainties are correlated for both transitions and therefore the covariance was included^77,78^ to propagate the uncertainty to the average $\text {5s}^2$21f $^2\text {F}_{5/2}$ value from both transitions. Transitions to the other principal numbers of the $\text {5s}^2n$f and $\text {5s}^2n$p series were then matched with the closest $\text {5s}^2$21f $^2\text {F}_{5/2}$ reference scans in time to determine the relative centroid shift of their hyperfine structure. These centroid shifts are presented in Tables 4 and 5 for the series measured using the $\text {5s}^2$5p $^2\text {P}_{3/2}$ $\rightarrow$ $\text {5s}^2$5d $^2\text {D}_{5/2}$ (325.6 nm) or $\text {5s}^2$5p $^2\text {P}_{3/2}$ $\rightarrow$ $\text {5s}^2$5d $^2\text {D}_{3/2}$ (325.9 nm) as first step transitions respectively. The centroid shifts for the $\text {5s}^2n$f and $\text {5s}^2n$p series were then used to determine their absolute energy levels using the absolute value for the $\text {5s}^2$21f $^2\text {F}_{5/2}$ state, as reported in Table 3.

**Table 3:** Determination of the absolute energy level of the $\text {5s}^2$21f $^2\text {F}_{5/2}$ reference state. The final value is the weighted mean from two sets of reference transition measurements, $\text {5s}^2$5d $^2\text {D}_{5/2}$ $\rightarrow$ $\text {5s}^2$21f $^2\text {F}_{5/2,7/2}$ and $\text {5s}^2$5d $^2\text {D}_{3/2}$ $\rightarrow$ $\text {5s}^2$21f $^2\text {F}_{5/2}$. ‘Lit.’ refers to the uncertainty on the lower state energy taken from literature^76^.

Transition	$\text {5s}^2$21f $^2\text {F}_{5/2}$	$\sigma$(Lit.)^76^	$\sigma (\nu )$	$\sigma$(Ext.)	$\sigma (\text {T}_\text {B})$	$\sigma (\lambda )$
	(MHz)	(MHz)	(MHz)	(MHz)	(MHz)	(MHz)
$\text {5s}^2$5d $^2\text {D}_{5/2}$ $\rightarrow$ $\text {5s}^2$21f $^2\hbox {F}_{5/2,7/2}$	1,391,645,930(185)	150	70	55	28	10
$\text {5s}^2$5d $^2\text {D}_{3/2}$ $\rightarrow$ $\text {5s}^2$21f $^2\hbox {F}_{5/2}$	1,391,645,782(173)	150	19	55	28	10
Average	**1,391,645,846(175)**					

Bold text indicate the final reported experimental and theoretical values.

The energy levels for the members of the $\text {5s}^2n$f $^2\text {F}_{5/2}$ and $\text {5s}^2n$f $^2\text {F}_{5/2, 7/2}$ series shown in Tables 4 and 5, and Fig. 4 have agreement between them, using the evaluated energy of the $\text {5s}^2$21f$^2\text {F}_{5/2}$ reference from Table 3. The few principal quantum numbers with available values in literature^79^, for the $\text {5s}^2n$f $^2\text {F}_{5/2, 7/2}$, $\text {5s}^2n$p $^2\text {P}_{1/2}$ and $\text {5s}^2n$p $^2\text {P}_{3/2}$ states, have agreement well within uncertainty.

**Table 4:** Energy levels of the $^2\text {F}_{5/2, 7/2}$ $\text {5s}^2n$f Rydberg series states determined from energy level shifts relative to the $^2\text {F}_{5/2, 7/2}$ $\text {5s}^2$21f reference state, alongside the quantum defects, $\delta _n$, of the levels. Statistical uncertainty is indicated in parenthesis. Systematic uncertainty from the reference state is indicated in braces.

Series	*n*	Centroid shift (MHz)	Energy level (cm$^{-1}$)	Literature energy level^61^ (cm$^{-1}$)	$\delta _n$
$^2\text {F}_{5/2,7/2}$ $\text {5s}^2n$f	12	− 15,510,479(20)	45,902.9328(6)[59]	45,902.92(22)	0.04010(4)
	13	− 12,098,644(10)	46,016.7393(5)[59]		0.04028(5)
	14	− 9,393,432(60)	46,106.976(2)[6]		0.04052(8)
	15	− 7,212,037(60)	46,179.739(2)[6]		0.0407(1)
	16	− 5,427,634(80)	46,239.260(3)[6]		0.0408(1)
	18	− 2,710,813(20)	46,329.8837(7)[59]		0.0406(1)
	20	− 769,186(6)	46,394.6494(2)[59]		0.0407(2)
	21	0	46,420.3070(5)[59]		0.0408(2)
	22	666,548(10)	46,442.5403(3)[59]		0.0408(2)
	23	1,247,917(400)	46,461.930(10)[6]		0.0408(9)
	24	1,758,074(100)	46,478.950(5)[6]		0.0407(6)
	25	2,208,136(60)	46,493.962(2)[6]		0.0406(5)
	26	2,607,211(20)	46,507.2739(7)[59]		0.0404(4)
	28	3,280,668(20)	46,529.7380(7)[59]		0.0403(5)

**Table 5:** Energy levels of the $^2\text {P}_{1/2}$ $\text {5s}^2n$p, $^2\text {P}_{3/2}$ $\text {5s}^2n$p and $^2\text {F}_{5/2}$ $\text {5s}^2n$f Rydberg series states determined from energy level shifts relative to the $^2\text {F}_{5/2}$ $\text {5s}^2$21f reference state, alongside the quantum defects, $\delta _n$, of the levels. Statistical uncertainty is indicated in parenthesis. Systematic uncertainty from the reference state is indicated in braces.

Series	*n*	Centroid shift (MHz)	Energy level (cm$^{-1}$)	Literature energy level^60^ (cm$^{-1}$)	$\delta _n$
$^2\text {P}_{1/2}$ $\text {5s}^2n$p	22	− 1,842,830(20)	46,358.8365(7)[59]		3.2238(2)
	23	− 922,829(20)	46,389.5244(6)[59]		3.2236(2)
	24	− 132,449(20)	46,415.8886(6)[59]	46,415.871(26)	3.2235(2)
	25	551,531(5)	46,438.7037(2)[59]		3.2233(2)
	26	1,147,394(6)	46,458.5796(2)[59]		3.2231(2)
	28	2,129,967(60)	46,491.355(2)[6]	46,491.338(26)	3.2229(2)
	30	2,900,676(60)	46,517.063(2)[6]	46,517.043(26)	3.2226(2)
$^2\text {P}_{3/2}$ $\text {5s}^2n$p	22	− 1,816,214(20)	46,359.7243(6)[59]		3.1969(2)
	24	− 112,789(7)	46,416.5444(2)[59]	46,416.526(26)	3.1966(2)
$^2\text {F}_{5/2}$ $\text {5s}^2n$f	16	− 5,427,114(30)	46,239.278(1)[59]		0.0404(1)
	18	− 2,711,018(10)	46,329.8768(4)[59]		0.0408(1)
	19	− 1,663,438(5)	46,364.8203(2)[59]		0.0409(1)
	20	− 769,307(10)	46,394.6453(4)[59]		0.0409(2)
	21	0	46,420.3070(5)[59]		0.0408(2)
	22	666,346(5)	46,442.5336(2)[59]		0.0411(2)
	23	1,247,803(10)	46,461.9289(5)[59]		0.0410(3)
	24	1,757,899(8)	46,478.9439(3)[59]		0.0410(3)
	25	2,208,075(100)	46,493.960(5)[6]		0.0407(7)
	26	2,607,218(100)	46,507.274(5)[6]		0.0404(7)
	27	2,962,711(20)	46,519.1321(7)[59]		0.0402(5)
	28	3,280,581(4000)	46,529.700(100)[6]		0.04(1)
	30	3,823,828(9)	46,547.8558(3)[59]		0.0401(6)
	32	4,268,023(10)	46,562.6726(4)[59]		0.0410(7)
	33	4,460,517(30)	46,569.093(1)[6]		0.0409(9)
	34	4,636,375(60)	46,574.959(2)[6]		0.040(1)
	38	5,205,941(60)	46,593.958(2)[6]		0.040(2)
	40	5,428,856(6)	46,601.3938(2)[59]		0.039(1)
	45	5,861,534(20)	46,615.8264(8)[59]		0.039(2)
	50	6,170,831(20)	46,626.1434(6)[59]		0.042(3)
	53	6,316,157(200)	46,630.991(5)[6]		0.037(7)
	54	6,359,037(200)	46,632.421(7)[6]		0.042(8)
	55	6,399,878(300)	46,633.780(10)[6]		0.04(1)
	57	6,475,198(50)	46,636.296(2)[6]		0.034(5)
	60	6,574,308(600)	46,639.600(20)[6]		0.03(2)
	61	6,603,688(70)	46,640.582(2)[6]		0.040(7)
	62	6,631,893(20)	46,641.5228(7)[59]		0.046(6)
	65	6,709,179(30)	46,644.101(1)[6]		0.048(7)
	68	6,776,687(200)	46,646.353(5)[6]		0.04(1)
	69	6,796,960(60)	46,647.029(2)[6]		0.05(1)
	70	6,816,857(100)	46,647.693(4)[6]		0.04(1)
	72	6,853,899(300)	46,648.928(9)[6]		0.03(2)

### Evaluation of the ionization potential of the indium atom

**Figure 5:**
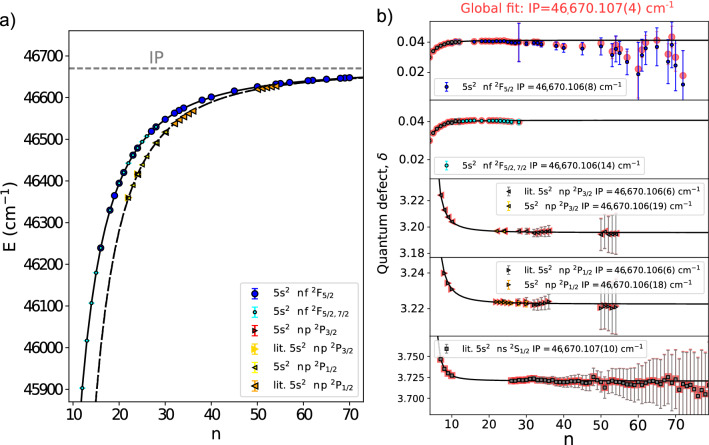
(**a**) Extracted energies of the $\text {5s}^2n$p $^2\text {P}_{1/2}$, $\text {5s}^2n$p $^2\text {P}_{3/2}$, $\text {5s}^2n\text {f}\,^2\text {F}_{5/2}$ and $\text {5s}^2n\text {f}\,^2\text {F}_{5/2, 7/2}$ Rydberg series measurements. Literature values, labeled as ‘lit.’, were taken from Ref.^60^. The black lines indicates the Rydberg expression values for the $^2\text {F}_{5/2, 7/2}$ $\text {5s}^2n$f (solid line) and $^2\text {P}_{1/2, 3/2}$ $\text {5s}^2n$p series (dashed line). The ionization potential is indicated by the dashed grey line. (**b**) Determination of the ionization potential of the indium atom using a global simultaneous fit using Expressions 6 and 7 to $\delta _n$ values of the $\text {5s}^2n$f $^2\text {F}_{5/2}$, $^2\text {P}_{1/2,3/2}$ $\text {5s}^2n$p, $^2\text {P}_{1/2,3/2}$ $\text {5s}^2n$p and $^2\text {P}_{1/2,3/2}$ $\text {5s}^2n$p of this work, along with literature (‘lit’) values for the $^2\text {P}_{1/2,3/2}$ $\text {5s}^2n$p series^60^ and $^2$S$_{1/2}$ $\text {5s}^2n$s^85^ series. The red markers indicate series $\delta _n$ values after the global fitting (IP = 46,670.107(4) cm$^{-1}$). The IP value determined from the fit to each series independently are shown in their corresponding subplots. The black lines indicate series $\delta _n$ values determined by Expression 7 from lowest-lying states^61^ (*n* = 4-10 for *n*f, *n* = 5-10 for *n*p and *n* = 6-10 for *n*s). Figure created using Refs.^44,45^.

The energy levels of the $^2\text {P}_{1/2}$ $\text {5s}^2n$p, $^2\text {P}_{3/2}$ $\text {5s}^2n$p, $^2\text {F}_{5/2}$ $\text {5s}^2n$f and $^2\text {F}_{5/2, 7/2}$ $\text {5s}^2n$f Rydberg series states determined in this work are shown in Fig. 2 in comparison to the accepted literature ionization potential (IP) of the indium atom^67^. The energies of the the $n^{\text {th}}$ Rydberg series states, E$_n$, can be determined using the Rydberg expression^80,81^

6 $$\begin{aligned} E_n = \text {IP} + \frac{R_{\text {115In}}}{(n - \delta _n)^2} \; , \end{aligned}$$

where $\delta _n$ is the quantum defect^82^, a measure of the difference in electronic structure for the Rydberg series of a multi-electron atom compared to hydrogen, included as the effective principal quantum number $n^* =$n$- \delta _n$. The effect due to the finite mass of $^{115}$In compared to the electron, is given by the Rydberg constant $R_{\text {115In}}$^81^ of $109736.79\,\hbox {cm}^{-1}$, which was derived from Penning trap atomic nuclei mass measurements^83,84^. Expression 6 can be fitted to the experimental energy levels, leaving the IP and $\delta _n$ as free parameters. The result of this is shown by the black lines in Fig. 5a. Expression 6 was fitted to lower-lying *n* states of the series^61^ to predict laser frequency scan ranges and give *n* assignments.

The $\delta _n$ values evaluated from our experimental energies are shown in Fig. 5b alongside the Ritz expansion values (black lines) from^86^

7 $$\begin{aligned} \delta _n = a_0 + \frac{a_1}{(n-\delta _0)^2} + \frac{a_2}{(n-\delta _0)^4} + \frac{a_3}{(n-\delta _0)^6} \; \; , \end{aligned}$$

where $a_{0,1,2,3}$ and $\delta _0$ are parameters fitted to measurements of energies of lower-lying states for each series from literature^76,87,88^. This gave the behaviour of the $\delta _n$ values for increasing *n*. The $a_{0,1,2,3}$ and $\delta _0$ values obtained for these series are given in Table 6. The value for the IP can be determined by using it as a free parameter to fit the $\delta _n$ values to this expression. The importance of measuring Rydberg states over a wide range of *n*, not just for high-lying *n* states, for fitting the IP with the $\delta _n$ values is clearly seen in Fig. 5 b, as the uncertainty in $\delta _n$ scales as ${n^*}^{-3}$. Furthermore, deviations in the experimental $\delta _n$ from that expected by Expression 7 can be used to identify deficiencies in the energy level measurements being used to determine the IP, due to a susceptibility to stray electric fields (in principle avoided by separation of the field ionization from the laser excitation step in a collinear setup) or perturbing configurations lying above the IP^89^. Large perturbations were found in the $\text {5s}^2n$d $^2$D series from the $^2$D term of the 5s5p$^2$ configuration^85^. No statistically significant deviation outside of the values of Expression 7 was observed within the accuracy of the $\text {5s}^2n\text {f}\,^2\text {F}_{5/2}$, $\text {5s}^2n\text {f}\,^2\text {F}_{5/2,7/2}$, $\text {5s}^2n$p $^2\text {P}_{1/2}$, $\text {5s}^2n$p $^2\text {P}_{3/2}$ series measurements performed in this work.

The $\delta _n$ values were fitted to Expression 7 for each Rydberg series to obtain the value for the IP independently for each. The resulting IP values are presented in Table 6 and in the sub plots of Fig. 5b. The IP values extracted from the series measurements and from literature are in good agreement. The $\delta _n$ obtained from the $\text {5s}^2n$p $^2\text {P}_{1/2,3/2}$ and $\text {5s}^2n$s $^2$S$_{1/2}$ series taken from literature^60,85^ are also shown in Fig. 5b and the resulting IP values in Table 6.

As the value of the IP is a common parameter for all of the series, a global simultaneous fit with the IP as a free parameter was performed using the $\delta _n$ values from the series measurements in this work in addition to individual $\delta _n$ values from literature. The shared and therefore correlated systematic uncertainties in the $\delta _n$ values were taken into account by using the covariance matrix of errors to weight the least squared fitting, performed using the Levenberg-Marquardt method^90,91^ as implemented in Refs.^92,93^. This yielded a combined value for the IP of $46,670.107(4)\,\hbox {cm}^{-1}$, an improvement over the previous highest precision literature value for the IP of $46,670.106(6)\,\hbox {cm}^{-1}$, derived from the $\text {5s}^2n$p $^2\text {P}_{1/2,3/2}$ series in Ref.^60^. In addition, our analysis took into account the correlated systematic uncertainty of $0.019\,\hbox {cm}^{-1}$ in the $\text {5s}^2n$p $^2\text {P}_{1/2}$ and $\text {5s}^2n$p $^2\text {P}_{3/2}$ energy levels of Ref.^60^. The levels used to determine the value of the IP in literature, in this work and in our global fit are indicated respectively by grey, coloured markers and red markers in Fig. 7. The difference of 0.2% of the theoretical IP from the experimental value is well within that expected under the RCSSD approximation^94^, in contrast to difference by a factor of 5 observed in the $A_{\text {hf}}$ constants. This further highlights the difference in electron correlation trends for the calculation of hyperfine structure constants, in contrast to energies, for the same RCSSD level of approximation.

**Table 6:** Values for the ionization potential of the indium atom evaluated by fitting the the quantum defects of the $\text {5s}^2n$f $^2\text {F}_{5/2}$ and $\text {5s}^2n$p $^2\text {P}_{3/2,1/2}$ Rydberg series, fitting to the series separately and simultaneously (‘sim. fit.’). The determined $a_{0,1,2,3}$ and $\delta _0$ parameters for Expression 7 are presented. Quoted IP uncertainties include statistical and systematic uncertainties propagated as described in the text.

Series	IP (cm$^{-1}$)	$\delta _0$	$a_0$	$a_1$	$a_2$	$a_3$
**Theory**						
DHF	41,507.11					
RCCSD	46,762.85					
$+$Breit	46,725.95					
$+$QED	**46,763.57**					
Literature						
*n*p $^2\text {P}_{1/2, 3/2}$, Ref.^60^	46,670.106(6)					
*n*s $^2$S$_{1/2}$, Ref.^85^	46,670.107(10)					
*n*f $^2\text {F}_{5/2, 7/2}$, Ref.^87^, 7$\le n \le$10	46,670.110(50)					
This work						
*n*f $^2\text {F}_{5/2}$	46,670.106(8)	0.04	0.041	− 0.153	0.714	− 2.296
*n*f $^2\text {F}_{5/2, 7/2}$	46,670.106(14)	0.04	0.041	− 0.153	0.714	− 2.296
*n*p $^2\text {P}_{3/2}$	46,670.106(19)	3.22	3.196	0.382	0.125	3.1454
*n*p $^2\text {P}_{1/2}$	46,670.106(18)	3.25	3.223	0.380	0.112	3.1258
**Global fit** (This work & Literature)	**46,670.107(4)**					

Bold text indicate the final reported experimental and theoretical values.

## An improved field-ionization setup

### Systematics of the field-ionization setup

In order to explore possible improvements for future spectroscopy studies, using the type of grid field-ionization arrangement presented in this work, a systematic study of the setup was performed. The energy spread of the ions created by field-ionization in the setup was found to be an important consideration. The spread in the position where the Rydberg atoms are ionized is determined by the ionization probability for the Rydberg atom in the electric field gradient created by the electric potential. Therefore the ionization probability in a given electric field gradient ultimately determines the spread in electric potential the ions are produced and the energy spread of the ion beam as it exits the ‘field-ionization region’. The situation of the Rydberg atom bunch encountering a step increase in electric field as they travel into ‘field-ionization region’ is equivalent to the application of a pulsed electric field to the Rydberg atoms at rest, which has been studied more extensively^95–97^. In the adiabatic limit where the classical electron motion is fast compared to the electric field pulse, the field necessary to reach saturation of ionization for the ensemble of Rydberg atoms (to ionize all Rydberg states above a given *n* within the pulse duration) is calculated^96^ to be $E_n - \text {IP} = -5.97F^{1/2}$ V/cm, corresponding to a field gradient of

8 $$\begin{aligned} F_{sat} = 3.38\times \frac{10^8}{n^{*4}} \; \; \text {V/cm}, \end{aligned}$$

using the parameters for an indium Rydberg atom. This is similar to the commonly used estimate for the critical field ionization strength in the case of a static electric field^57,98^ of $F_{crit} \approx 3\times 10^8/n^{*4} \; \text {V/cm}$. The classical Kepler period^97^ of

9 $$\begin{aligned} \tau _K = 2\pi \frac{m_e a_0^2}{\hslash } n^{*3} \; \; , \end{aligned}$$

for an electron in the $\text {5s}^2$70f $^2\text {F}_{5/2}$ state is $\tau _K$=52 ps, where $a_0$ is the Bohr radius and $m_e$ the electron mass. This can be used to estimate the cutoff for the adiabatic limit. The distance within which Rydberg atoms can be assumed to be ionized applying the electric field gradients according to Expression 8, is then given by $l_{sat}=\tau _K\nu _B$, for an atom beam of velocity $\nu _B$. In the case of atomic $^{115}$In at $T_\text {B}$ = 25 keV this corresponds to 10.65 $\upmu \hbox {m}$. This results in a minimum energy spread of $\Delta E$ = 8 eV for the electric gradient of *F*=7.5 $\mathrm{kV\,cm}^{-1}$ used in this work ($\Delta E=l_{sat}F$). The corresponding time-of-flight broadening for this minimum energy spread is well below the 2 $\upmu \,\hbox {s}$ FWHM ion bunch width from the ablation ion source and was not resolvable in this work. In order to go below this intrinsic energy spread, higher electric field gradients would be required to ensure ionization in a short distance, although scaling as $F \propto n^{*-2}$ appears in the sub-ps regime^97^.

**Figure 6:**
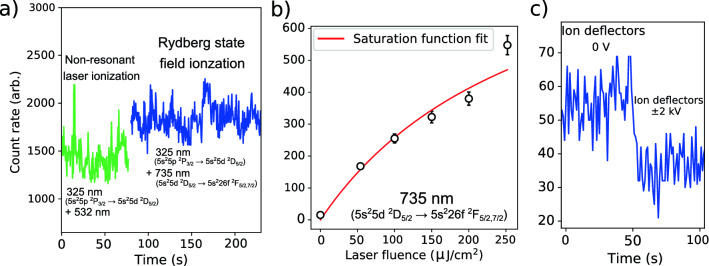
(**a**) The count rate on resonance for a typical non-resonant photoionization last step versus the field-ionization scheme corresponding to their total ionization efficiencies, (**b**) fitting the saturation curve^99^ of the $\text {5s}^2\text {5d}\,^2\text {D}_{3/2}$ $\rightarrow$ $\text {5s}^2$26f $^2\text {F}_{5/2,7/2}$ transition, giving a saturation fluence of 293(140) $\mu$J/cm$^2$ and (**c**) background rates with and without electrostatic deflectors to estimate background in the ‘interaction’ and ‘post-ionization’ region. Figure created using Refs.^44,45^.

Figure 6a shows the detected ion-count rate of a measurement performed using the $\text {5s}^2$5p $^2\text {P}_{3/2}$ $\rightarrow$ $\text {5s}^2\text {5d}\,^2\text {D}_{5/2}$ (325.6 nm) first step followed by non-resonant ionization using 532-nm light produced by a Litron TRLi HR 120-200 Nd:YAG laser. This was recorded in order to make a comparison with the total ionization efficiency of the field-ionization setup, using the same first step followed by the $\text {5s}^2\text {5d}\,^2\text {D}_{3/2}$ $\rightarrow$ $\text {5s}^2$26f $^2\text {F}_{5/2,7/2}$ transition and field ionization. The measurements were performed less than a minute apart following optimization of the overlap of the 735-nm, 532-nm and 325-nm light with the neutral atoms, aligned using two irises before and after the interaction region of the beamline (as indicated in Fig. 1). The laser pulses were overlapped in time using a photodiode at the laser exit window of the beamline. A maximum output pulse fluence of 55 mJ/cm$^{2}$ was used for the 532-nm step, with no discernible decrease in count rate observed down to 44 mJ/cm$^{2}$. Meanwhile the $\text {5s}^2\text {5d}\,^2\text {D}_{3/2}$ $\rightarrow$ $\text {5s}^2$26f $^2\text {F}_{5/2,7/2}$ transition appeared not to be saturated as indicated in Fig. 6b, with a maximum of 250(20) $\mu$J/cm$^2$ available and the estimated saturation fluence of 293(140) $\mu$J/cm$^2$. A scatter of around 10% in beam intensity was due to shot-to-shot variations intrinsic to the ablation ion source setup used^31,100^. While this makes an exhaustive study difficult, this underlines two issues under these typical measurement conditions: (i) It was more difficult in practice to ensure the same level of efficiency with a non-resonant laser ionization scheme than a saturated field-ionization setup. This could be due to the non-linear nature of non-resonant photoabsorption into the continuum^101^ or reduced efficiency of collecting and detecting ions from a larger volume or re-neutralisation of the ions (at pressures of around $1\times 10{-9}$ mbar this can still give an appreciable contribution^29^). (ii) A larger laser fluence is required in order to saturate transitions to high *n* Rydberg states such as the $\text {5s}^2\text {5d}\,^2\text {D}_{3/2} \rightarrow$ $\text {5s}^2$26f $^2\text {F}_{5/2,7/2}$ transition, as the transition strength decreases with *n*^102^. An appropriate *n* Rydberg state has to be chosen to ensure saturation of the transition in addition to a electric field gradient to ensure ionization according to Expression 8. This is an additional validation of the fact that the technique lends itself well to use on bunched atomic beams, where high laser fluence pulsed lasers can be used.

In order to study the factor of reduction in collisional background using the field-ionization setup shown in Fig. 3, measurements were performed at pressures raised by a factor of 10 compared to the nominal operating level, increasing the signal for the collisional background rate. The pressure in the ‘interaction’ region of length $l_1=$120 cm was raised to $5\times 10^{-9}$ mbar ($\rho _1=1.2\times 10^{14}\,\hbox {m}^{3}$), and the ‘post-ionization’ region of length $l_2=$30 cm was raised to $5\times 10^{-8}\hbox {mbar} (\rho _2=1.2\times 10^{15}\,m^{-3})$.

For a measured neutral beam current of $I_B=6.0(1)\times 10^{6}$ atoms/s, and a collisional ion beam current, I$_C$, the cross section for collisional ionization can be defined as

10 $$\begin{aligned} \sigma = \frac{I_{C}}{I_B \rho l} \; \; \text {cm}^2 \; . \end{aligned}$$

As both regions will have the same value of $\sigma$, measurements of the collisional ion currents can be used as a consistency check for the reduction in ionization volume using the known atom path lengths, *l*, and residual gas densities, $\rho$. The remaining background ion current from applying ±2 kV electrostatic deflectors in the ‘field-ionization’ region gave the collisional ion current for the ‘post-ionization’ region, while applying the ground potential gave the background ion current from both ‘interaction’ plus ‘post-ionization’ regions. The measured ion currents were I$_{C}^{l_2}$ = 35(5) ions/s and I$_{C}^{l_1+l_2}$ = 55(5) ions/s, respectively, as shown in Fig. 6c. From these measurements the collisional ionization cross sections for the indium atom incident upon residual gas atoms at 25 keV were determined to be $\sigma _1 =2.3(8)\times 10^{-16}\,\hbox {cm}^{2}$ and $\sigma _2=1.62(23)\times 10^{-16}\,\hbox {cm}^{2}$ for the ‘interaction’ and ‘post-ionization’ regions, respectively. The larger error of $\sigma _1$ results from taking the difference between I$_{C}^{l_2}$ and $\hbox {I}_{C}^{l_1+l_2}$ to determine I$_{C}^{l_1}$. The cross sections are in agreement and are of the expected order of magnitude at a beam energy of 25 keV^103^. This demonstrates a consistency for a factor of five in length (and volume assuming a homogeneous beam diameter) reduction (from 150 cm to 30 cm, as indicated in Fig. 1) for the source of collisional background ions. In addition this shows that the largest source of remaining atom-beam related background is due to ions created by collisional ionization with residual gases in the ‘post-ionization’ region, which are not able to be removed by the electrostatic deflectors in the ‘field-ionization’ region. The background suppression of the design in this work is therefore limited by the length of the ‘post-ionization region’ and the vacuum pressure in that region.

The simulated electric field gradient of Fig. 3c highlights an additional consideration when using parallel wires for field ionization. The approximation of a planar electric potential breaks down as the wires are approached and inhomogenities in the penetrating field create a large spike in the experienced electric field gradient. This property is in fact useful for defining the point of ionization and reducing the ion energy spread, however the potential geometry of Fig. 3b creates three positions where the electric field gradient is greatest and approximately equal in magnitude. It is therefore crucial for the critical field for ionization saturation to be applied to avoid ionization across more than one position which would result in a maximum energy spread of the magnitude of the potential applied.

### Energy selective electrode design

**Figure 7:**
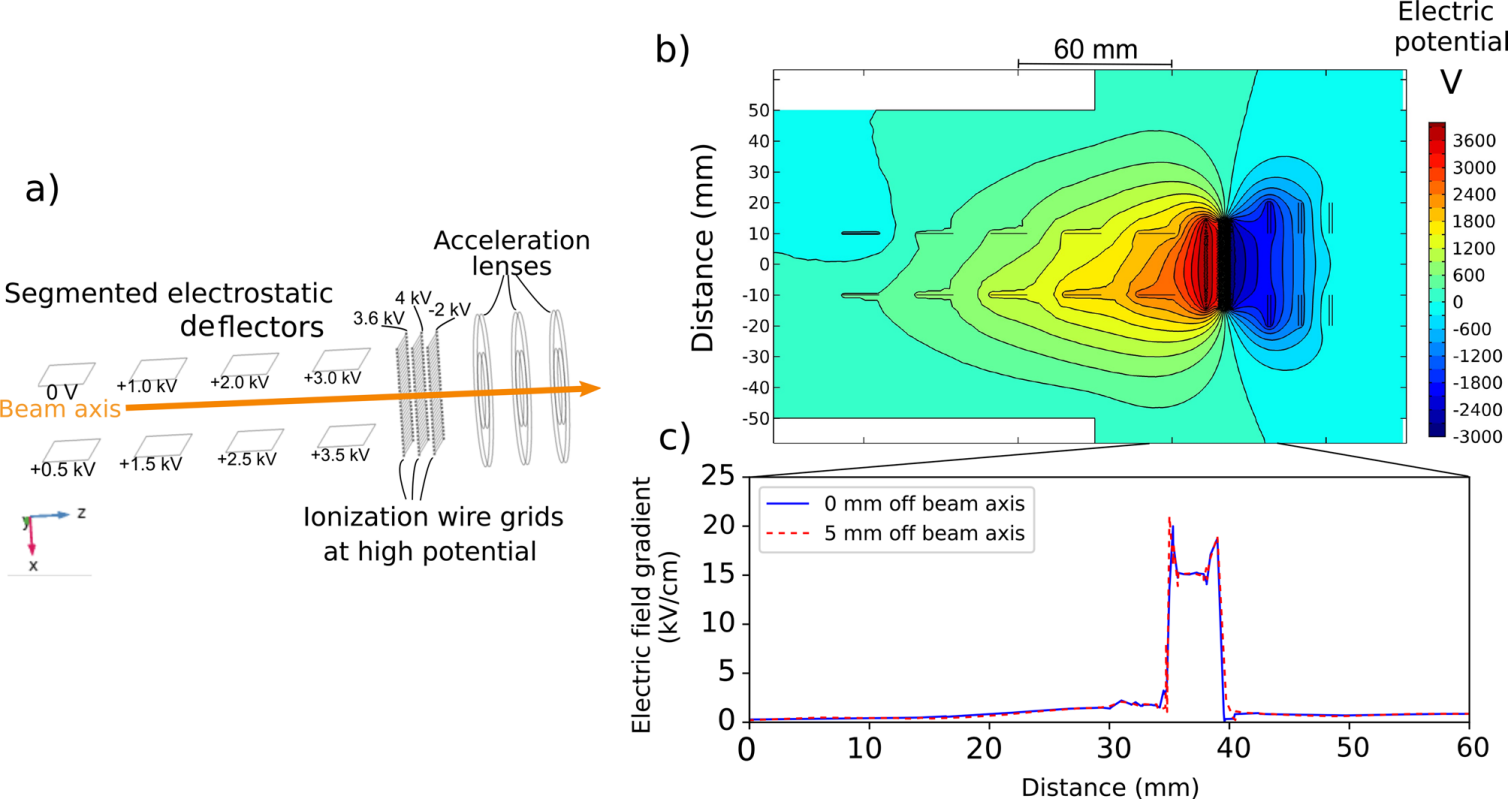
(**a**) A schematic of the improved electrode arrangement simulated from this work (**b**) The electric potential of an optimised field-ionization setup, and (**c**) the accompanying electric field gradient, simulated using voltages compatible with a 25 keV beam. Figure created using Refs.^44,45,56^.

Although the design used in this work effectively removes background from collisional ions created in the ‘interaction’ region before the ‘field-ionization’ region, the remaining background from ions created in the ‘post-ionization region’ can still be substantial. With this consideration, an improved design has been developed to detect only those ions created inside the small volume of the field-ionization grids, allowing a 1:1 correspondence between the factor of 300 or greater reduction in ionization volume and background suppression. The improved design is shown in Fig. 7. The principle of the design is to create an energy shift for the ions created in the ‘field-ionization’ region. This introduces energy selectivity for the Rydberg states ionized in the ‘field-ionization’ region, distinguishing them from other background sources of ions which will remain at the initial beam energy. Compared to the design used for the measurements in this work, this removes the demand for a short ‘post-ionization region’ with the best possible vacuum conditions.

**Figure 8:**
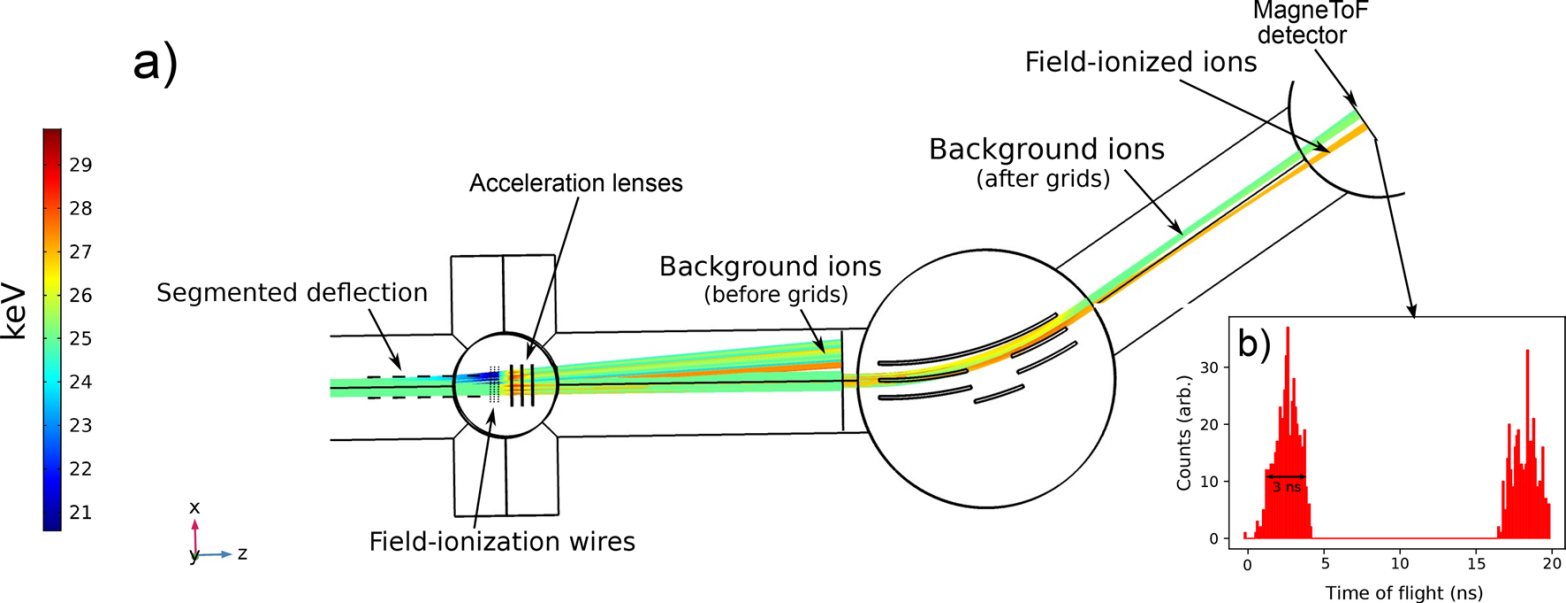
(**a**) The simulated ion trajectories using the field-ionization arrangement shown in Figure 7 and (**b**) the corresponding simulated time-of-flight difference for ions from field ionization versus background. Figure created using Refs.^44,45,56^.

In this improved design, the opposite polarity deflector plates (Fig. 3a,b) are replaced by segmented flat electrodes^57^ of the same polarity, but with a potential difference of around 500 V between them to provide the equivalent electrostatic deflection of background ions created before the field-ionization grids (Fig. 8a,b). These electrodes are labelled as “segmented electrostatic deflectors” in Fig. 7a. Electrostatic lenses are also included following the field-ionization grids, labelled as “acceleration lenses” in Fig. 7a. These additional ion optic elements were designed with simple planar geometries to be compatible with fabrication using metal traces on printed circuit boards^104^.

This design allows the outer grids to be held at a higher and adjustable electric potential without compromising the advantages of the previous field-ionization arrangement. The segmented electrodes allow the Rydberg atoms to enter a high potential without an abrupt increase in electric field gradient causing ionization. The removal of the opposite polarity deflector plates allows the potential of the first grid to be raised without introducing a large asymmetry in the electric potential or a large electric field gradient transverse to the atom beam axis. In addition, this creates a well defined electric field gradient without the need for outer grounding grids. The principle of this arrangement is to reduce the electric field gradient between the first and second grids, moving the step to high electric field gradient to the middle grid instead. The step can be made greater by applying an opposite polarity potential to the last grid, as shown in Fig. 7. This localizes the field-ionization region to a raised potential, resulting in an increase in beam energy of Rydberg states which are ionized in this potential. The ‘acceleration lenses’ following the field-ionization grids, are then used for extraction from the raised potential.

The resulting ion trajectories from this ionization arrangement are shown in Fig. 8a, where the increase in beam energy of ions created inside the field-ionization region is indicated. The ions with the beam energy of interest can be selectively detected following electrostatic deflection, because the deflection introduces an angular separation of ions with different energies, as seen in Fig. 8a. Using slits (or a position sensitive detector) to select ions of a given beam energy, allows the ions created by field ionization to be distinguished from any other background source of ions created in the ‘interaction’ or ‘post-ionization’ regions. This not only includes collisional ions, but ions created by field ionization of collisionally excited or re-neutralised atoms in the field of the 20$^{\circ }$ bend to the ion detector, photoionization or molecular breakup, as all of these sources of background ions will remain at the lower beam energy. Alternatively the beam energy could be measured directly^105^, or the difference in detected time of flight of the ions could be used as a gate if the bunch width was sufficiently narrow. For example, the time of flight separation introduced in the ‘post-ionization’ region for the ions travelling at 25 keV is around 15 ns (Fig. 8b), so a bunch window narrower than this would be needed. The incident temporal atom bunch width of $2\,\upmu \mathrm{s}$ (FWHM) in this work would prevent this. The beam energy difference for ions created by field ionization could be enhanced by using lower incident beam energies or higher potential for the ionization apparatus, however the design of the ion optics then becomes more critical to avoid ion transmission loses.

The improved field-ionization design outlined here therefore offers improved background suppression over the design used for measurements in this work, by providing selectivity of ions created by field ionization independent of the length and vacuum quality of the ‘post-ionization’ region. In general, the background suppression factor for the improved field-ionization design compared to non-resonant laser ionization can be expressed as

11 $$\begin{aligned} S_{BG} = \frac{L}{l_{ion}} \frac{P_L}{P_l} \; \; . \end{aligned}$$

where *L* is the path length of the ‘interaction’ plus ’post-ionization’ regions, $l_{ion}$ is the path length in which ionization can take place inside the ‘field-ionization’ region, and $P_L/P_l$ is the ratio vacuum pressure in the two regions. This is under the approximation of a homogeneous gas composition in the regions and a uniform atomic beam diameter. The energy selectivity offers the prospect of a reduction in ionization volume by a factor of $1.6\times 10^{5}$, down from a region of length $L=150\,\hbox {cm}$ to the $l_{ion}=10.65\,\upmu \hbox {m}$ for the adiabatic cut-off assumed in the field-ionization model of Expression 8, where $l_{ion}=l_{sat}=\tau _K\nu _B$. However the electrostatic bend used in the CRIS experimental setup, combined with adjustable slits to select an ion path incident on the detector has an energy resolution of around $\sigma _ E$ = 1.5 keV, which can only guarantee a selectivity of the ionization volume down to $l_{ion}=F$/$\sigma _E$. For the value of $F~=~7.5\,kV\,\hbox {cm}^{-1}$ used in this work, this corresponds to a volume reduction by a factor of $1.25\times 10^{3}$. Below this limit, direct beam energy measurement, or ion time-of-flight measurement using ion bunches narrower than 15 ns would be necessary to determine the actual energy spread and confirm the precise background suppression factor.

When combined with extreme-high vacuum technologies^106^ to improve the vacuum quality in the field-ionization region (increase the $P_L/P_l$ ratio), this technique has the potential to reduce the dominating collisional background ion contribution to a vanishingly low level when compared to other sources of background, such as non-resonant ionization from the lower pulse energy resonant step laser light, the dark count rate of the detector ($\sim$0.08 cps for an ETP DM291 MagneTOF), or residual radioactivity in the setup.

## Conclusion

The use of ion cooling and bunching has allowed highly sensitive measurements of exotic atoms and molecules containing short-lived isotopes to date^1,3^, by concentrating measurements on ion bunches into a narrow time window, in order to improve background suppression and additionally allowing a high duty cycle for high-resolution and high-detection efficiency pulsed laser ionization spectroscopy^20,42,43^.

In this work we have implemented field ionization with the Collinear Resonance Ionization Spectroscopy (CRIS) technique, to further increase the selectivity (and thus sensitivity) of high-resolution measurements of hyperfine spectra of isotopes in atom bunches. This allows the ionization to take place in a narrow spatial window in addition to the narrow time window, substantially reducing background due to collisional ions created alongside the atoms of interest in larger ionization volumes. Here we have demonstrated a factor of five in ionization volume reduction and corresponding background suppression, when accounting for vacuum pressure. In principle this will allow measurements of exotic isotopes with yields down to 4 atoms per second at the CRIS experiment. However, a further factor of >400 improvement in background suppression of collisional ionization shown to be possible with an improved design, which also makes background suppression independent of distance from field ionization to ion detection by incorporating an increase in beam energy of the field-ionized Rydberg atoms. Furthermore, as a non-resonant pulsed laser step is no longer necessary to ionize the atom bunches, this removes a significant source of photo-ionization background, in addition to removing a source of AC Stark shifts in measurements from short-lived metastable states^55^.

By using bunched atomic beams the technique is well suited to the use of narrow-band pulsed lasers, taking advantage of the high spectral density to saturate transitions to high-lying Rydberg states required for field ionization. The $\text {5s}^2n$p $^2$P and $\text {5s}^2n\text {f}\,^2$F Rydberg series states in the indium atom up to *n* = 72 were studied and used to evaluate the ionization potential of the indium atom to be $46,670.107(4)\,\hbox {cm}^{-1}$, in agreement with, and improving upon the precision of previous measurements. Furthermore, the technique allows high resolution measurements of the hyperfine structure constants and isotope shifts of individual atomic states directly.

The nuclear magnetic dipole, nuclear electric quadrupole hyperfine structure parameters and isotope shifts of the $^{113}$In and $^{115}$In isotopes, for the $\text {5s}^2$5d $^2\text {D}_{5/2}$ and $\text {5s}^2$5d $^2\text {D}_{3/2}$ states were measured. The experimental results were compared to DHF, RCCSD and AR-RCCSD calculations, where a good level of agreement was found with experimental isotope shifts and the ionization potential of the indium atom. While the RCCSD calculations showed an improvement over DHF calculations for the $\text {A}_\text {hf}$ constants, the magnitudes were underestimated, indicating that electron correlations play a crucial role in these $\text {5s}^2\text {5d}\,^2\text {D}_{5/2}$ and $\text {5s}^2\text {5d}\,^2\text {D}_{3/2}$ states, demanding further theoretical study.

Improvements in highly sensitive detection techniques compatible with precise laser spectroscopy are required to measure the nuclear structure of the most exotic nuclei produced at radioactive beam facilities, important for developing nuclear theories^36,38,40,107–110^. In addition, it has many potential applications, such as the separation of nuclear waste^5^, enrichment of nuclear fuel^11^, collection of nuclear isomers^9^, “ultra”-trace analysis^7^, research of nuclear-spin-dependent effects^8,10^ and highly-purified nuclear decay spectroscopy^6^.
